# EEG feature selection method based on maximum information coefficient and quantum particle swarm

**DOI:** 10.1038/s41598-023-41682-5

**Published:** 2023-09-04

**Authors:** Wan Chen, Yanping Cai, Aihua Li, Yanzhao Su, Ke Jiang

**Affiliations:** Rocket Force University of Engineering, Xi’an, 710025 China

**Keywords:** Biomedical engineering, Applied mathematics

## Abstract

To reduce the dimensionality of EEG features and improve classification accuracy, we propose an improved hybrid feature selection method for EEG feature selection. First, MIC is used to remove irrelevant features and redundant features to reduce the search space of the second stage. QPSO is then used to optimize the feature in the second stage to obtain the optimal feature subset. Considering that both dimensionality and classification accuracy affect the performance of feature subsets, we design a new fitness function. Moreover, we optimize the parameters of the classifier while optimizing the feature subset to improve the classification accuracy and reduce the running time of the algorithm. Finally, experiments were performed on EEG and UCI datasets and compared with five existing feature selection methods. The results show that the feature subsets obtained by the proposed method have low dimensionality, high classification accuracy, and low computational complexity, which validates the effectiveness of the proposed method.

## Introduction

The electroencephalogram (EEG) is a signal that records changes in scalp potential. It contains rich brain activity and behavioral cognition information and is often used for brain activity analysis and disease diagnosis^1^. EEG-based disease diagnosis, emotion analysis, and mental state monitoring are pattern recognition problems^2^. To improve the recognition accuracy, the number of channels for EEG acquisition equipment is increasing^3^. Meanwhile, the types of EEG features are constantly enriched^4^, which leads to an exponential increase in the dimension of EEG features. However, uncorrelated features will affect the classification accuracy, and high-dimensional features will increase the computational load^5^. Therefore, extracting an effective feature subset is beneficial to improve the classification accuracy of EEG and reduce the computational cost.

Principal component analysis (PCA) is a data dimensionality reduction technique widely used in the dimension reduction of EEG features^6^. However, the physical meaning of the feature subset obtained after PCA dimensionality reduction is far from the original feature^7^. By eliminating redundant features, the feature selection method can obtain the optimized feature subset, improving the classification accuracy without changing the meaning of features, and is conducive to EEG classification^8,9^. Depending on the evaluation criteria, feature selection methods can be classified into two types: Filter and Wrapper. The filter feature selection method is independent of the subsequent learning algorithm. It directly sorts the importance of all features through a specific scoring criterion and extracts the top-ranked features to form feature subsets^10^. Cai et al.^11^ adopted the Minimal redundancy maximal relevance (MRMR) method to achieve EEG feature extraction. Tuncer et al.^12^ adopted the fusion method of ReliefF and iterative neighborhood component analysis (RFINCA) to achieve EEG feature extraction. All the above methods reduce the feature dimension and improve the classification accuracy to some extent. However, these methods do not consider the overall performance of feature subsets, and their contribution to classification accuracy is limited^13^. The Wrapper feature selection method uses the classification error rate as the evaluation criterion for feature subsets and is therefore highly accurate. For example, Jiang et al.^14^ used the genetic algorithm and support vector machine (SVM) to select and classify EEG features and obtained high recognition accuracy. However, the Wrapper feature selection is a global search algorithm, and it is easy to fall into local optima in high-dimensional feature selection, which affects the classification accuracy in the later stage^15^. With the increase of EEG feature dimension and the improvement of recognition accuracy requirements, the above feature selection method has been unable to meet the actual needs^16^. Therefore, in this paper, we hybridize the two feature selection methods to improve the feature selection ability of the proposed algorithm in high-dimensional data.

Existing hybrid feature selection methods can be divided into two steps^17^. First, a filter feature selection method is used to remove some features to reduce the search space in the second step, and then an optimization algorithm is used to optimize the feature subset^18^. Therefore, hybrid feature selection methods can achieve higher classification accuracy and reduce the feature space dimensionality by combining the advantages of filter feature selection and wrapped feature selection^19^. Song et al.^20^ proposed a fast hybrid feature selection method based on correlation-guided clustering and particle swarm optimization (PSO). This approach divides feature selection into three stages. In the first and second stages, filter feature selection and clustering algorithms are used to reduce the search space in the third stage. Then, in the third stage, PSO is used to find the optimal subset. Ansari et al.^21^ proposed a hybrid filter-wrapper feature selection method for sentiment classification. In this approach, the feature subset is first selected from the feature set by a rank-based feature selection method, and then the optimal feature subset is selected by PSO. Xue et al.^22^ proposed a fault feature selection algorithm combining the ReliefF and quantum particle swarm optimization (QPSO). First, ReliefF is used to classify features and remove irrelevant ones. The features are then further selected using QPSO to obtain the optimal subset of features. These methods have shown good performance on datasets in their respective domains. However, EEG datasets show characteristics of small sample size and high feature dimensionality, which are different from common datasets^23^. Therefore, it remains to be verified whether these methods are suitable for EEG feature selection. In the filtering feature selection stage, many methods only deal with irrelevant features and ignore redundant features, which can affect the feature optimization effect in the second stage^24,25^. Moreover, in the stage of feature subset optimization based on the evolutionary algorithm, traditional fitness functions are mainly based on the classification accuracy of the test set, ignoring the importance of dimensionality reduction^26^.

To address the above issues, a hybrid feature selection method is proposed for EEG feature selection. In the first stage, the method uses MIC to deal with irrelevant and redundant features, which effectively reduces the search space in the second stage. On the one hand, irrelevant features are eliminated by computing the relationship between features and categories. On the other hand, redundant features are eliminated by computing the correlation between the selected features and the features to be selected. In the second stage, we design a new fitness function that combines classification accuracy and dimensionality reduction rate to improve classification accuracy while minimizing feature dimensionality. The feature subset is then further optimized using QPSO to obtain the optimal feature subset. Finally, SVM is used for EEG classification. Meanwhile, considering the interaction between feature subset and classifier, the proposed method synchronously optimizes feature subset and SVM parameters in the second stage to improve classification accuracy.

## Related work

### Maximum information coefficient

MIC is a correlation measurement method based on information theory, and its scope of application and accuracy is superior to other correlation measurement methods such as Pearson correlation coefficient, mutual information, and information gain^13^. Meanwhile, it has low computational complexity and is suitable for measuring the correlation between various features. For two random sequences $D = \left\{ {(f_{1,i} ,\;f_{2,i} ),\;i = 1,\;2,\; \ldots ,\;n} \right\}$, the MIC is defined as follows:

1 $$mic(D) = \mathop {\max }\limits_{XY < B} M(D)_{X,Y} = \mathop {\max }\limits_{XY < B} \frac{\max (I(D,X,Y))}{{\log_{2} (\min (X,Y))}}$$

where $X$ represents dividing the domain of $f_{1}$ into $X$ segments. $Y$ represents dividing the domain of $f_{2}$ into $Y$ segments. $XY < B$ represents the range of values for the number of grids. $I(D,X,Y)$ represents the mutual information of $D$ under $X$ columns and $Y$ rows. Since the row and column divisions are not equidistant, various meshing methods exist. $\max$ and $\min$ represent the maximum and minimum values. Reference^27^ pointed out that the effect is best when $B = n^{0.6}$, where $n$ represents the data length, so this paper also takes this value.

### Quantum particle swarm optimization

Sun et al.^28^ introduced quantum behavior into particle swarm optimization and proposed the QPSO algorithm, which improved the global search ability of particle swarms. Meanwhile, the QPSO algorithm only has a position vector, so it has fewer control parameters and stronger optimization ability. The implementation process of QPSO is as follows:

- Step 1: initialization.
  2 $$x^{j} = x_{\min }^{j} + (x_{\max }^{j} - x_{\min }^{j} ) \times rand(1,\;N)$$

where $x^{j}$ represents the j-th dimension of the particle. $x_{\max }^{j}$ and $x_{\min }^{j}$ represents the upper and lower limits of the j-th dimension. $N$ represents the size of the particle swarm.

- Step 2: Calculate $Pbest$ and $Gbest$.
  3 $$Pbest = x$$
  4 $$Gbest = x_{k} \, [x_{k} ,\;k] = \min (f(x))$$
  where min represents the minimum value, and $f$ represents the fitness function.

- Step 3: Update the position of the particle.
  5 $$P_{i,j} = \varphi_{j} \cdot Pbest_{i,j} + (1 - \varphi_{j} ) \cdot Gbest_{j}$$
  6 $$m_{best} = \frac{1}{N}\sum\limits_{i = 1}^{N} {P_{i} }$$
  7 $$\alpha = 0.5 + 0.5 \cdot (T_{\max } - T)/T_{\max }$$
  8 $$x_{i,j} (t + 1) = \left\{ \begin{gathered} P_{i,j} - \alpha \cdot |m_{bestj} - x_{i,j} (t)| \cdot \ln (1/u_{i,j} )\;\;r \ge 0.5 \hfill \\ P_{i,j} + \alpha \cdot |m_{bestj} - x_{i,j} (t)| \cdot \ln (1/u_{i,j} )\;\;r < 0.5 \hfill \\ \end{gathered} \right.$$
  where $i$ represents the particle and $j$ represents the dimension. $x(t)$ represents the t-th generation particle. $\varphi$, $u$, and $r$ are random numbers in the (0,1) interval. $\alpha$ is called the contraction expansion coefficient. $T$ represents the current iteration number. $T_{\max }$ represents the maximum number of iterations.

- Step 4: Update $Pbest$ and $Gbest$.
  9 $$Pbest_{i} = \left\{ \begin{gathered} x_{i} \, (f(x_{i} ) < f(Pbest_{i} ) \hfill \\ Pbest_{i} \, otherwise \hfill \\ \end{gathered} \right.$$
  10 $$Gbest = \left\{ \begin{gathered} x_{k} \, (f(x_{k} ) < f(Gbest)) \hfill \\ Gbest \, otherwise \hfill \\ \end{gathered} \right.$$
  where $x_{k}$ represents the particle with the smallest fitness value.

- Step 5: Determine whether the termination condition is met. If not, return to Step 3. Otherwise, output the optimal value.

### Support vector machine

The SVM can obtain the optimal global solution by using the principle of structural risk minimization as the optimality principle. It performs well on small sample nonlinear problems. Moreover, it has the advantages of simple system structure, global optimization, strong generalization ability, and short learning and prediction time when dealing with classification problems. Therefore, it is often used to solve various classification problems^29,30^. The basic idea of SVM is to map the input data to a high-dimensional space so that the input data is linearly separable. The core problem is to solve the minimization problem:

11 $$\begin{gathered} \mathop {\min }\limits_{w,b} \;\frac{1}{2}\left\| w \right\|^{2} + C\sum\limits_{i = 1}^{n} {\xi_{i} } \hfill \\ s.t.\;\;y_{i} (w^{T} x^{(i)} + b) \ge 1 - \xi_{i} ,\;\xi_{i} \ge 0 \hfill \\ \end{gathered}$$

where $w$ and $b$ represent the weight vector and bias of the hyperplane. $C$ represents the regularization parameter. $\xi$ represents the slack variable. $y_{i}$ represents the category of the i-th experiment. $x^{(i)}$ represents the feature vector of the i-th experiment. When the data is converted from low-dimensional to high-dimensional space, the kernel function is necessary, and the most commonly used kernel function is the Gaussian kernel function:

12 $$K_{g} (x,\;z) = \exp \left( { - \frac{{\left\| {x - z} \right\|^{2} }}{{2\sigma^{2} }}} \right)$$

where $\sigma$ represents the bandwidth of the kernel function. The regularization parameter $C$ and kernel function bandwidth $\sigma$ significantly influence the performance of SVM, and the parameters can be optimized by the grid method or optimization algorithm. The proposed method uses the classification error rate of SVM to evaluate the feature subset, so there is a correlation between the parameters of the SVM and the feature subset. Therefore, QPSO is used to optimize the parameters of the feature subset and SVM synchronously to obtain a better classification effect.

## EEG feature

Many scholars have conducted meaningful studies in EEG analysis and proposed a large number of reliable EEG features. In this paper, five commonly used EEG features are extracted, including approximate entropy (ApEn), power spectral density (PSD), Hjorth parameter, CO complexity, and fractal dimension (FD).

1. Approximate entropy.

Approximate entropy (ApEn) is a nonlinear dynamic parameter used to quantify the regularity and unpredictability of time series fluctuations. ApEn uses a non-negative number to represent the complexity of a time series, reflecting the possibility of new information appearing in the time series. The more complex the time series, the larger the approximation entropy. For a time series consisting of N data, the M-dimensional reconstruction of the signal is first performed:

13 $$X_{m} (i) = \left\{ {x(i),\;x(i + 1),\; \ldots ,\;x(i + m - 1)} \right\},\;1 \le i \le N - m + 1$$

The distance between $X_{m} (i)$ and $X_{m} (j)$ is defined as:

14 $$d[X_{m} (i),\;X_{m} (j)] = \mathop {\max }\limits_{k = 0,\; \ldots ,\;m - 1} (|x(i + k) - x(j + k)|)$$

Given a threshold value r, for each i, count the number of $d \le r$, and calculate the ratio of $B_{i}$ to $N - m - 1$:

15 $$B_{i}^{m} (r) = \frac{1}{N - m - 1}B_{i}$$

Then average the logarithm of $B_{i}^{m} (r)$:

16 $${\Phi }^{{\text{m}}} ({\text{r}}) = ({\text{N}} - {\text{m}} + 1)^{ - 1} \mathop \sum \limits_{{{\text{i}} = 1}}^{{{\text{N}} - {\text{m}} + 1}} \log ({\text{B}}_{{\text{i}}}^{{\text{m}}} ({\text{r}}))$$

Finally, the ApEn of the signal can be estimated as:

17 $$ApEn = \Phi^{m} (r) - \Phi^{m + 1} (r)$$

(2) Power spectral density.

PSD is used to describe the change of signal power with frequency. In this paper, we used the periodogram to estimate the PSD, and its mean value was used as the EEG feature. The Fourier transform of signal x is X, then the spectral characteristics of the signal are calculated as follows:

18 $$f_{PSD} = \frac{1}{N}\sum {|X|}^{2}$$

(3) Hjorth parameter.

The Hjorth parameter describes the signal characteristics in terms of Activity, Mobility and Complexity. For the signal x, the Hjorth parameter is calculated as follows:

19 $$Activity = \frac{1}{N}\sum\limits_{n = 1}^{N} {(s(n) - \mu_{s} )^{2} }$$

20 $$Mobility = \sqrt {\frac{{{\text{var}} (s^{\prime}(n))}}{{{\text{var}} (s(n))}}}$$

21 $$Complexity = \frac{Mobility(s^{\prime}(n))}{{Mobility(s(n))}}$$

where $\mu_{s}$ represents the average value, ${\text{var}}$ represents the variance, and $s^{\prime}(n)$ represents the first derivative.

(4) CO complexity.

CO complexity is used to describe the irregularity of the signal. The signal can be divided into regular and irregular parts, and CO complexity defines the ratio of irregular parts. It reflects the complexity and randomness of the signal. For a signal x(n), the Fourier transform is first performed on the signal, and then the average value of the power spectrum is calculated:

22 $$M = \frac{1}{N}\sum\limits_{k = 0}^{N - 1} {|X(k)|^{2} }$$

where $X(k)$ represents the fast Fourier transform of x(n), and N represents the length of the signal in the frequency domain. Then a new spectral sequence is constructed using M and X(k) :

23 $$Y(k) = \left\{ \begin{gathered} X(k) \, |X(k)|^{2} > M \hfill \\ 0 \, |X(k)|^{2} \le M \hfill \\ \end{gathered} \right.$$

Finally, the CO complexity of signal is obtained:

24 $$CO = \frac{{\sum\limits_{n = 0}^{N - 1} {|x(n) - y(n)|^{2} } }}{{\sum\limits_{n = 0}^{N - 1} {|x(n)|^{2} } }}$$

where y(n) is the inverse Fourier transform of Y(k).

(5) Fractal dimension.

Fractal dimension (FD) can characterize the complexity of time domain signals. For a time-domain signal x(t) of length N, the signal is first transformed as follows:

25 $$X_{\tau }^{T} = \left\{ {x(\tau ),\;x(\tau + T),\; \ldots ,\;x\left( {\tau + \left[ {\frac{N - \tau }{T}} \right]} \right)} \right\}$$

where $\tau = 1,2,...,T$. For the transformed signal X, its length sequence is defined as:

26 $$L_{\tau } (T) = \frac{1}{T}\frac{{\sum\limits_{i = 1}^{{\left[ {\frac{N - \tau }{T}} \right]}} {|x(\tau - iT) - x(\tau (i - 1)T)| \times (N - 1)} }}{{\left[ {\frac{N - \tau }{T}} \right]}}$$

Then calculate the average length of each sequence:

27 $$L(T) = \mathop \sum \limits_{\tau = 1}^{T} L_{\tau } (T)$$

All values from $T_{\min }$ to $T_{\max }$ are calculated, and an average length sequence is obtained. Then the slope of linear fitting for $\ln L(T)$ to $\ln L(1/T)$ is estimated as the fractal dimension of the signal.

## Improved feature selection method

The Filter method obtains feature subsets by ranking the quality of individual features^10^, thus the quality for each feature is well. However, the classification results rely on feature subsets rather than individual features. The Filter method lacks the evaluation of the overall performance for the feature subset, resulting in low accuracy^17^. The Wrapper method uses the classification accuracy of the classifier as the overall evaluation metric for the feature subset, and thus the classification accuracy is higher than that of Filter. However, the Wrapper method has a higher computational load than the Filter method, and when the feature dimension is high, the dimension of the obtained feature subset is relatively high^18,20^. In this paper, the proposed method first uses MIC to remove irrelevant and redundant features and then uses QPSO and SVM for quadratic feature selection, which can better eliminate irrelevant and redundant features while ensuring classification accuracy.

### MIC-based feature pre-selection

In this paper, MIC is used to eliminate irrelevant and redundant features. First, the correlation between the feature vector and the category vector is measured by MIC. Sort the feature vectors in descending order by correlation. Features whose correlation is less than a certain threshold are irrelevant features. Then, the correlation between feature vectors is measured by MIC. Features whose correlation is greater than a certain threshold are redundant features.

For an nm-dimensional feature matrix $X = \left\{ {x_{i} ,\;i = 1,\;2,\; \ldots ,\;m} \right\}$, an n-dimensional category vector $y = \left\{ {y_{i} ,\;i = 1,\;2,\; \ldots ,\;n} \right\}$, where n represents the number of samples and m represents the number of features. Denote the correlation between feature $x_{i}$ and $x_{j}$ as $mic(x_{i} ,\;x_{j} )$, and the correlation between feature ${\varvec{x}}_{i}$ and category $y$ as $mic(x_{i} ,\;y)$. The value of MIC ranges from 0 to 1. The larger the value, the greater the correlation between the data. $mic > 0.8$ indicates a strong correlation between the data. $mic < 0.2$ indicates a weak correlation between the data ^13^. $mic(x_{i} ,\;x_{j} )$ measures the correlation between features. When $mic(x_{i} ,\;x_{j} )$ is larger than 0.8, it indicates that the correlation between the two features is high. $mic({\varvec{x}}_{i} ,{\varvec{y}})$ measures the correlation between features and categories. When $mic(x_{i} ,\;y)$ is less than 0.2, it indicates that the correlation between features and categories is weak. Therefore, in this paper, we set the threshold to 0.8 for redundant features and 0.2 for irrelevant features.

Then, the implementation steps of MIC-based feature pre-selection are as follows:

- Step 1: For feature vectors $x_{i} ,\;i = 1,\;2,\; \ldots ,\;m$ and category vector $y$, compute their maximum information coefficients $mic(x_{i} ,\;y),\;i = 1,\;2,\; \ldots ,\;m$.
- Step 2: When $mic(x_{i} ,\;y)$ is less than 0.2, $x_{i}$ is an irrelevant feature, and then remove $x_{i}$ from the feature matrix.
- Step 3: For the remaining feature vectors, calculate the MIC between different feature vectors $mic(x_{i} ,\;x_{j} ){\kern 1pt} ,\;i \ne j$.
- Step 4: When $mic(x_{i} ,\;x_{j} )$ is greater than 0.8, compare the values of $mic(x_{i} ,\;y)$ and $mic(x_{j} ,\;y)\;$. if $mic(x_{i} ,\;y)\; < mic(x_{j} ,\;y)$, then $x_{i}$ is a redundant feature, otherwise $x_{j}$ is a redundant feature.
- Step 5: Remove all redundant features, and finally get a pre-selected feature subset.

### Secondary feature selection based on QPSO

After removing irrelevant and redundant features through MIC, the dimensionality of the original features will be significantly reduced. After that, the Wrapper method is used for quadratic feature selection, which can further optimize the performance of feature subsets and optimize the SVM parameters. There are two critical issues in feature selection using QPSO: the mapping between particles and features and the design of the fitness function.

For the first question, it should be emphasized that QPSO optimizes both the feature subset and the SVM parameters. Thus, the dimension m of each particle is equal to the dimension of a pre-selected subset of features plus the number of SVM parameters to be optimized. Among them, the first m-2 dimensions of the particle correspond to the dimension of the feature subset, respectively, and the value range is (0, 1). When the value is (0, 0.5), the dimension is not selected, and when it is [0.5, 1), it means that the dimension is selected. The last two dimensions of the particle correspond to the parameters of the SVM and have a range of positive values.

**Figure 1:**
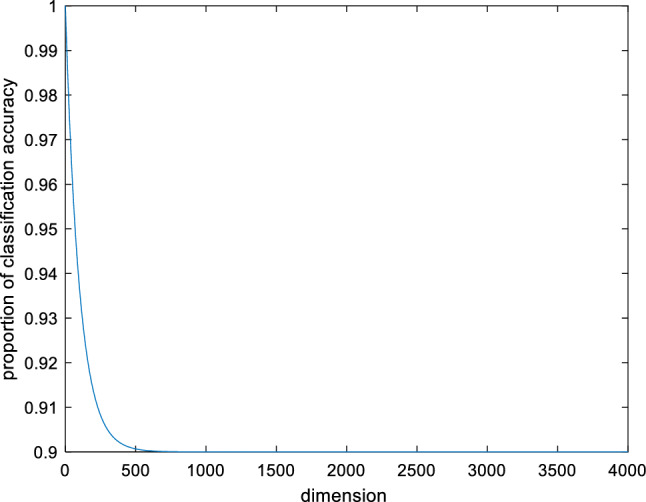
Transformation curve of proportion for classification accuracy.

For the second problem, the classification accuracy rate of the classifier is often used as the fitness function of the optimization algorithm, but there are limitations in using the classification accuracy only as an evaluation metric. It ignores the effect of dimensionality reduction. Especially when dealing with high-dimensional data, selecting fewer features is also of practical interest. Therefore, in this paper, we construct a new fitness function based on SVM recognition accuracy and dimensionality reduction rate:

28 $$f(X) = \varphi CA + (1 - \varphi )DR$$

29 $$\varphi = \frac{9 + \exp ( - d/100)}{{10}}$$

where $CA$ represents the classification accuracy, $DR$ represents the dimension reduction. $d$ represents the original feature dimension. $\varphi$ represents the proportion of classification accuracy. According to Eq. (29), the relationship curve between feature dimension and classification accuracy can be obtained, as shown in Fig. 1.

The Fig. 1 shows that the value approaches 1 when the feature dimension is low, indicating that the fitness function considers classification accuracy almost exclusively. This value gradually decreases as the feature dimension increases until it approaches 0.9, indicating that the fraction of classification accuracy gradually decreases while the fraction of dimension reduction gradually increases. The new fitness function takes into account both classification accuracy and dimensionality reduction. In particular, as the feature dimension increases, the fraction of dimension reduction also increases to some extent, which is more in line with the actual needs. The specific implementation steps for feature selection based on QPSO are as follows:

- Step 1: Initialize the number of particles $N$, the particle swarm $X$, and the maximum number of iterations $T_{\max }$.
- Step 2: The feature matrix and SVM parameters are extracted based on the particles and the SVM classifier is used for data classification. During model training, K-fold cross-validation is used to improve the performance of the model. According to Eqs. (28) and (29), the fitness value of the particle is obtained.
- Step 3: Initialize the optimal historical value and the optimal global value according to Eqs. (3) and (4).
- Step 4: Let $t = 1$, and update the particle position according to Eqs. (5)–(8).
- Step 5: Update the optimal historical value and the optimal global value according to Eqs. (9) and (10).
- Step 6: Let $t = t + 1$, when $t \le T_{\max }$, return to Step 4. Otherwise, output the optimal global value, and obtain the accurately selected feature subset.

### EEG feature selection method

The algorithm is summarized as follows:
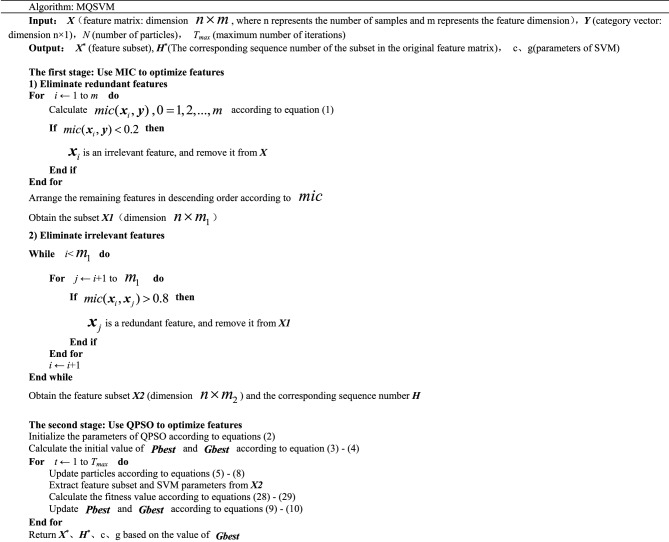


## Experimental results and analysis

### EEG dataset

**Figure 2:**
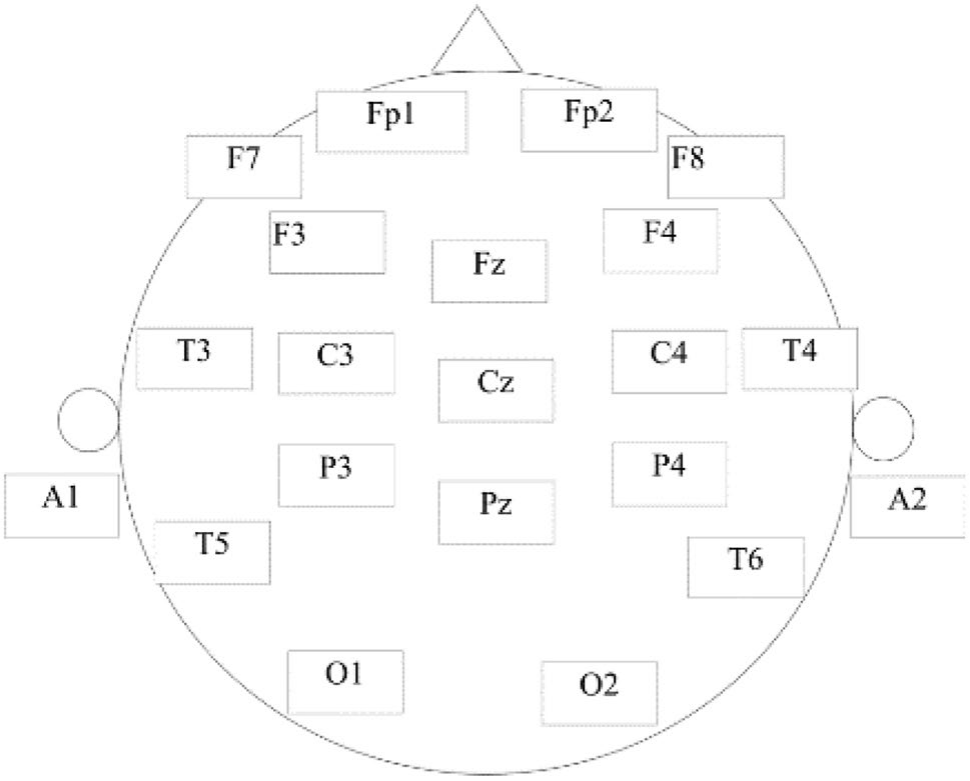
Electrode placement position.

A public data set provided by Mumtaz et al. was utilized to evaluate the proposed method^30^. A total of 64 subjects participated in the experiment, including 34 MDD subjects (17 men, average age 40.3 ± 12.9) and 30 normal control (NC) subjects (21 male, average age 38.23 ± 15.64), all participants are from Hospital Universiti Sains Malaysia (HUSM). MDD participants with psychotic symptoms, alcoholism, smoking and epilepsy were excluded from the study. The healthy control group was also excluded for possible mental or physical illness. All participants signed an informed consent form and were informed of the details of the trial. The experiment was designed in accordance with the Helsinki Declaration and approved by the HUSM Ethics Committee. The 19-channel EEG cap was used for EEG recording, and the electrodes were placed in the international standard 10–20 system, including Fp1, F3, C3, P3, O1, F7, T3, T5, Fz, Fp2, F4, C4, P4, O2, F8, T4, T6, Cz and Pz, as shown in Fig. 2, where A1 and A2 were the reference electrodes.

The resting-state EEG signals were acquired from MDD and HC subjects in the eye-opened (EO) condition. These signals are sampled at 256 HZ and filtered with a 0.5 Hz to 50 Hz bandpass filter and an additional 50 Hz notch filter. The artifacts were then removed using EEGLAB. Subjects 14 and 25 in the NC group and 7, 8, 12, and 34 in the MDD group were excluded because the EEG was less than 4 min after processing. Therefore, the data used in this study came from 58 subjects, including 28 NC and 30 MDD. Meanwhile, the EEG of the middle four minutes was intercepted to reduce the effect of noise at both ends. In order to improve the sample size, each subject's data were segmented according to 10 s, and finally, a total of 1392 (58 × 24) samples were obtained.

### EEG features

The sampling frequency of EEG signals is 256HZ, so six layers of wavelet transform are designed to extract delta, theta, alpha, beta, and gamma bands. On this basis, the Hjorth parameter, approximate entropy (ApEn), power spectral density (PSD), fractal dimension (FD), and CO complexity were extracted from each frequency band. Finally, the features of all channels and frequency bands are combined as feature vectors. Table 1 shows the specific information of EEG features. The number of signal channels is 19, and the signal of each channel is decomposed into five frequency bands, so the dimension of the feature is 95 (19 × 5). The Hjorth parameter contains three perspectives, so its dimension is 285 (95 × 3).

**Table 1:** Details of EEG features.

Number	Features	Dimension
1	CO	95
2	ApEn	95
3	FD	95
4	Hjorth	285
5	PSD	95
6	Fusion feature	665

### Ablation study

To verify the effectiveness of the proposed method, an ablation experiment was conducted, and the experimental setup is shown in Table 2. MQSVM1, MQSVM2, and MPSVM are hybrid feature selection methods, FS-QPSO is Wrapper feature selection, and FS-MIC is Filter feature selection. Five-fold cross-validation was used in the experiments. Five-fold cross-validation is to divide the data into five parts, each time extracting one part (not repeated) as the verification set, the remaining four parts as the training set. The training set is used to train the model. Also, three-fold cross-validation is used in the model training, that is, the k value of the proposed method is set to 3. The trained model is then applied to the validation set and the experimental results are obtained. The above operation is repeated five times and the results are averaged to obtain the final result. MPSVM uses PSO to optimize feature subsets and classifier parameters. The two acceleration coefficients of the PSO are set to 2, the inertia weight to 1, and the number of iterations and particles to 200. Other methods use QPSO to optimize feature subsets or classifier parameters. The number of iterations and particles in the QPSO is set to 200. FS-MIC uses a forward search strategy to find the optimal subset. All experiments were performed on Intel(R) Xeon(R) CPU, 2.3 GHz, 128 GB RAM.

**Table 2:** Comparison methods of experiments.

Method	The difference between the method and the proposed method
MQSVM1	Proposed method
MQSVM2	Replace the fitness function of QPSO with classification accuracy
MPSVM	Replace QPSO with PSO
FS-QPSO	Only QPSO was used for feature selection
FS-MIC	Only MIC was used for feature selection

**Figure 3:**
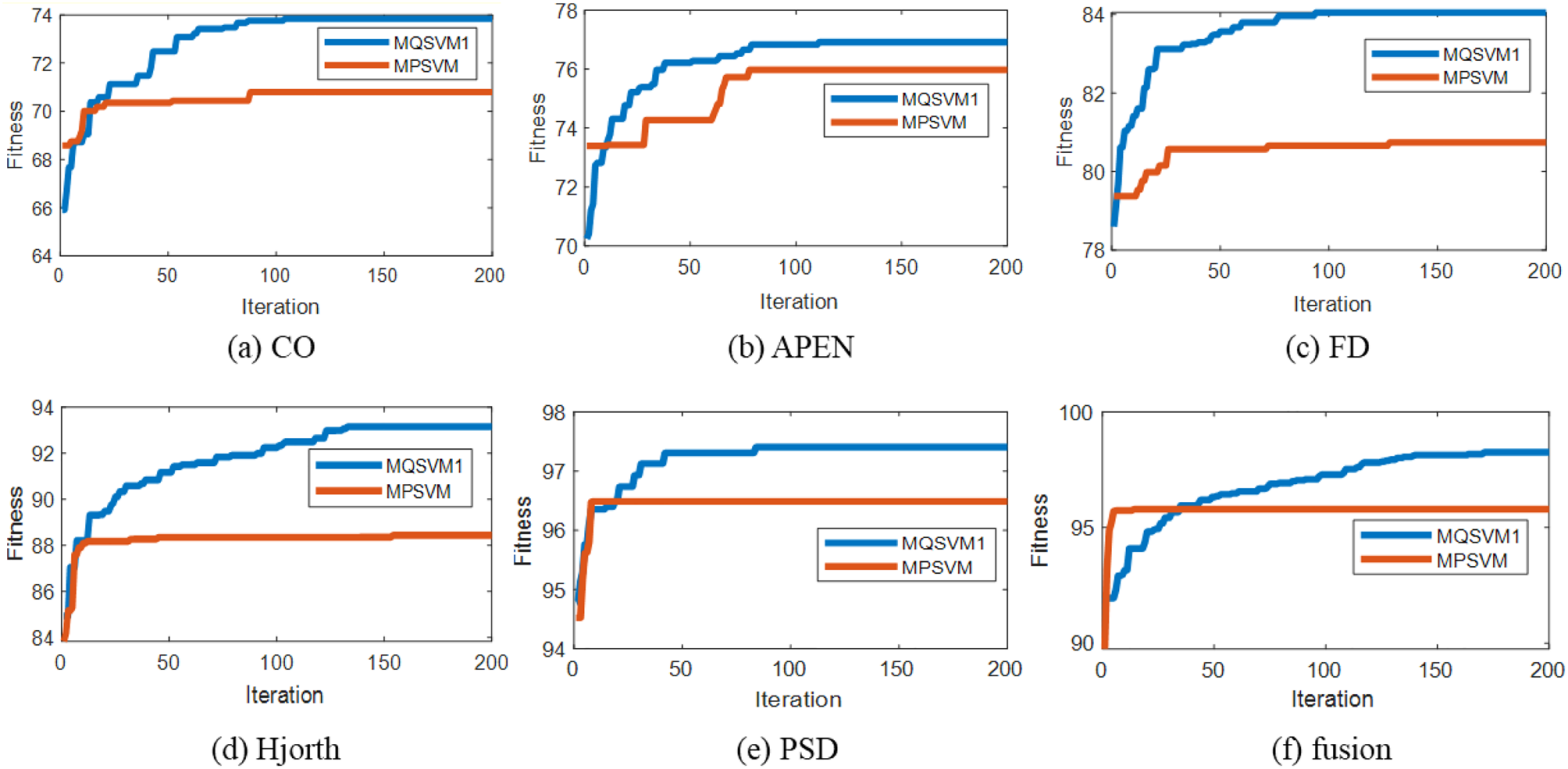
The change curve of fitness value in the second stage.

Figure 3 shows the optimization process of MQSVM1 and MQSVM2 in the second stage. It can be seen that the fitness values of MQSVM1 and MQSVM increase with the number of iterations. Meanwhile, the fitness value reaches the convergence value before the end of the iteration, indicating that the particle number and iteration number settings are valid. The convergence value of MQSVM2 is lower than that of MQSVM1 for different EEG features because PSO tends to get stuck in local optima, while QPSO can jump out of local optima. The above analysis shows that the performance of the proposed method is higher when QPSO is used in the second stage.

Table 3 shows the running times of all methods. It can be found that the running time of all methods increases with the feature dimension. The running time of FS-MIC increases significantly with the feature dimension, since FS-MIC employs a forward search strategy, which requires the QPSO to optimize the classifier parameters each time a feature is accepted. The running time of MQSVM1 gently increases with the feature dimension, since MQSVM1 uses QPSO to optimize the classifier parameters while optimizing a subset of features. When the feature dimension is low (CO, APEN, FD), the running time of FS-MIC is small, which is due to the low computational complexity of the Filter method. When the feature dimension is high (fusion), the running time of MQSVM1 is small, indicating that the proposed method is suitable for high-dimensional data sets. In terms of average running time, FS-QPSO has the longest running time, while the other four methods have almost no difference in running time, indicating that the Wrapper method has the highest computational complexity. In particular, the running time of FS-QPSO is very long in high-dimensional features, which is not favorable for practical applications. The above analysis shows that the proposed method is not computationally complex and can be used for EEG feature selection.

**Table 3:** Runtime of different methods (min).

Feature	MQSVM1	MQSVM2	MPSVM	FS-QPSO	FS-MIC
CO	223.90	276.21	181.86	392.20	**101.05**
APEN	183.04	225.86	225.23	394.90	**101.13**
FD	143.08	193.26	128.90	371.91	**100.46**
Hjorth	344.23	**289.89**	294.15	503.57	349.12
PSD	**123.41**	163.77	126.79	143.00	200.84
Fusion	**344.36**	388.35	309.81	1214.53	653.59
Mean	227.00	256.22	**211.12**	503.35	251.03

Bold values indicate the minimum runtime.

Table 4 shows the dimensions of the feature subsets. It can be seen that the feature subset dimension after feature selection is significantly lower than the original feature dimension. The dimension of feature subsets obtained by three hybrid feature selection methods (MQSVM1, MQSVM2, MPSVM) is significantly lower than that of the Wrapper and the Filter feature selection methods, which indicates that the hybrid feature selection method has advantages in feature selection. Among all EEG features, the dimensionality of the feature subset obtained by the proposed method is significantly lower than that of the other two hybrid feature selection methods, indicating that the proposed improved strategy is effective. The dimensionality of the feature subset of MQSVM1 is significantly lower than that of MQSVM2, which indicates that the fitness function constructed in this paper using the dimensionality of the feature subset and the classification accuracy is effective. The dimension of the feature subset of MQSVM1 is significantly lower than that of MPSVM, indicating that PSO is more likely to get stuck in the local optimal solution when optimizing the feature subset. At the same time, QPSO can jump out of the local optimal solution, so the dimension of the feature subset obtained by MQSVM1 is low. The above analysis shows that the dimension of the feature subset obtained by the proposed method is lower than that of the other methods, which is beneficial for reducing the computational complexity of the proposed method.

**Table 4:** Dimension of the feature subset.

Feature	Dimensionality	MQSVM1	MQSVM2	MPSVM	FS-QPSO	FS-MIC
CO	95.00	**11.40**	25.80	20.40	50.80	32.00
APEN	95.00	**11.80**	29.40	28.20	51.40	32.00
FD	95.00	**11.40**	29.40	16.60	51.80	32.00
Hjorth	285.00	**27.00**	88.80	54.60	114.40	106.20
PSD	95.00	**13.80**	48.60	21.40	33.80	62.80
Fusion	665	**46.20**	141.60	87.00	303.60	183.40
Mean	221.67	**20.27**	60.60	38.03	100.97	74.73

Bold values indicate the smallest dimension of the feature subset.

Table 5 shows the classification accuracy of EEG. It can be found that the classification accuracy of FS-QPSO is significantly higher than the other methods because the Wrapper method can evaluate the overall performance of feature subsets, which is beneficial for improving the classification accuracy of feature subsets. The classification accuracy of MQSVM1 is significantly higher than that of FS-MIC, which indicates that the proposed method can combine the advantages of the Wrapper method and improve the classification accuracy of the proposed method. The classification accuracy of MQSVM1 is higher than that of MPSVM, which indicates that QPSO has more optimization power than PSO, and therefore it is more advantageous to use QPSO for feature optimization.

**Table 5:** Classification accuracy of EEG.

Feature	MQSVM1	MQSVM2	MPSVM	FS-QPSO	FS-MIC
CO	69.97	69.61	68.83	**70.83**	70.48
APEN	**74.28**	72.99	74.21	74.00	73.86
FD	82.55	83.76	80.31	83.98	**84.35**
Hjorth	**94.62**	93.72	89.51	**94.62**	91.37
PSD	**97.49**	97.20	96.41	97.41	96.70
Fusion	99.28	97.94	96.95	**99.62**	97.06
Mean	86.36	85.87	84.37	**86.74**	85.64

Bold values indicate the highest classification accuracy.

The weighted values of feature subset dimension and classification accuracy were calculated according to Eq. (28), and the results are shown in Table 6. The weighted values synthesize the dimensionality and classification accuracy of a subset of features, which facilitates direct comparison and analysis of the performance of different methods. The larger the weighted value, the better the performance of the feature subset obtained by the method. It can be found that the weighted value of FS-MIC is significantly lower than that of the other methods, indicating that the feature subset obtained by the Filter feature selection method performs relatively poorly. The weighted values of MQSVM1, MQSVM2, and FS-QPSO are higher, which indicates that the feature subsets obtained by the hybrid feature selection method and the Wrapper feature selection method have better performance. The weighted value of MPSVM is significantly lower than that of the other two hybrid feature selection methods, indicating that QPSO performs significantly better than PSO in feature subset optimization. In several EEG features (CO, APEN, Hjorth, PSD, fusion), MQSVM1 was weighted higher than other methods. Considering the average values, the weighted value of MQSVM1 is the largest, indicating that the feature subset obtained by the proposed method performs the best.

**Table 6:** The weighted value of feature subset dimension and classification accuracy.

Feature	MQSVM1	MQSVM2	MPSVM	FS-QPSO	FS-MIC
CO	**71.01**	69.80	69.38	69.44	70.24
APEN	**75.04**	72.76	73.99	72.39	73.42
FD	82.86	82.92	80.44	81.78	**83.31**
Hjorth	**94.24**	91.42	88.71	91.41	88.73
PSD	**96.80**	94.43	95.32	95.53	93.10
Fusion	**98.66**	96.03	95.95	95.10	94.61
Mean	**86.43**	84.56	83.97	84.27	83.90

Bold values indicate the largest weighted values of feature subset dimension and classification accuracy.

The proposed method runs faster when only MIC is used for feature selection. However, since the Filter feature selection method uses a forward search approach, the running time in high-dimensional datasets is extended when the classifier needs to optimize parameters. Therefore, the running time of Filter feature selection methods combined with some classifiers that do not require parameter optimization will be significantly reduced. In addition, the feature dimension and accuracy obtained by the Filter feature selection method are lower than those of Wrapper and hybrid feature selection methods because the feature subset obtained by the Filter feature selection method lacks the overall evaluation of the feature subset, so the performance of the feature subset obtained by the Filter feature selection method is not high. FS-QPSO achieves the highest classification accuracy, but its running time is longer than Filter and hybrid feature selection methods due to the ample search space. When the feature dimension is higher (fusion), the running time increases significantly, reaching 1214 min, which makes the method difficult in practical application. Therefore, although the feature subset obtained by the Wrapper feature selection method performs well, the computational complexity is too high, and it is not easy to apply it directly to high-dimensional features. MQSVM1 combines the advantages of Filter and Wrapper feature selection methods, and its runtime is significantly lower than FS-QPSO. The proposed method optimizes the parameters of the classifier while optimizing a subset of features in the second stage, which reduces the computational complexity of the method in high-dimensional features to some extent. Therefore, the running time of the proposed method is significantly slower than that of FS-QPSO as the dimension of EEG features increases. There are differences between MQSVM1 and MQSVM2 in terms of fitness function. MQSVM1 uses a fitness function constructed from feature subset dimension and classification accuracy, and MQSVM2 uses a fitness function constructed from classification accuracy. Experimental results show that the feature subset dimension of MQSVM1 is significantly lower than that of MQSVM2, and the classification accuracy of MQSVM1 is also high, which indicates that the fitness function designed in this paper can effectively reduce the dimension of the low feature subset and achieve high classification accuracy. MQSVM1 and MPSVM have different optimization algorithms. MQSVM1 uses QPSO to optimize feature subsets, and MPSVM uses PSO to optimize feature subsets. Experimental results show that the classification accuracy of MQSVM1 is significantly higher than that of MPSVM, which indicates that QPSO has a more robust search capability and can significantly reduce the probability of the method falling into local optimality. Therefore, it is efficient to use QPSO in the second stage to optimize the feature subset. In terms of weighted values, MQSVM1 achieves significantly higher values than the other methods, which verifies the effectiveness of the proposed improved strategy.

### Comparison with existing methods

To thoroughly verify the superiority of the proposed method, we compared the proposed method with other classical methods, including EEG feature selection methods and other domain feature selection methods. These include the MRMR EEG feature selection method adopted by Cai et al. in 2018^11^, the Fscore-based EEG feature selection method adopted by Wu et al. in 2018^24^, and the RFINCA EEG feature selection method adopted by Tuncer et al. in 2021^12^, the ReliefF-QPSO feature selection method adopted by Xue Rui et al. in 2020^15^, and the Fscore-MIC feature selection method adopted by Zhao Ling et al. in 2021^13^.

The experimental setup is the same as in Section “Ablation study”. The experiments are performed with five-fold cross-validation, the model is trained with three-fold cross-validation, and the number of particles and iterations of the QPSO is set to 200. The number of neighbors for ReliefF-QPSO and RFINCA is set to six. For the other methods, no other parameters need to be set. MRMR, Fscore, FSCOre-MIC, and RFINCA use a forward search strategy to find the optimal subset.

Table 7 shows the running times of the different methods. It can be found that as the feature dimension increases, the running time of all methods increases. MQSVM and Relief-QPSO are hybrid feature selection methods, but the running time of MQSVM is significantly lower than that of Relief-QPSO, mainly because ReliefF only deals with irrelevant features when performing feature selection. The proposed method uses MIC to deal with irrelevant and redundant features, which reduces the search space in the second stage and hence the computational complexity of the method. The remaining four methods are Filter feature selection methods, which have significantly lower running times than hybrid feature selection methods in low-dimensional features. However, the running time of the Filter feature selection method increases significantly in high-dimensional features, mainly because the Filter method uses a forward search method to find the optimal subset of features. Compared with existing methods, the running time of the proposed method grows more slowly with the increase of feature dimension, mainly because in the second stage, the proposed method optimizes the parameters of the classifier while optimizing the feature subset. The average runtime of MQSVM is 227 min, which is not a significant increase over existing methods. The above analysis shows that the computational complexity of the proposed method is not very high compared to the existing methods, and it increases slowly in high-dimensional features.

**Table 7:** Runtime of different methods (min).

Feature	MQSVM	ReliefF-QPSO	MRMR	Fscore	Fscore-MIC	RFINCA
CO	223.90	294.51	137.48	191.91	153.48	**99.10**
APEN	183.04	289.02	90.55	150.78	134.59	**84.91**
FD	143.08	152.70	131.96	155.55	137.29	**72.10**
Hjorth	344.23	427.18	234.27	380.80	328.35	**182.03**
PSD	**123.41**	179.17	124.76	210.84	127.59	216.38
Fusion	344.36	754.38	**246.28**	555.03	644.12	360.10
Mean	227.00	349.49	**160.89**	274.15	254.24	169.10

Bold values indicate the minimum runtime.

Table 8 shows the dimensions of the feature subsets. It can be found that the dimensionality of the feature subsets obtained by all methods is significantly reduced. The dimension of the feature subset obtained by Relief-QPSO is significantly higher than that obtained by the other methods. In high-dimensional features (Hjorth, fusion), the performance of Relief-QPSO is significantly reduced, which is because the fitness function of Relief-QPSO does not consider the influence of the feature subset dimension, and the ReliefF only processes irrelevant features, resulting in a large search space in the second stage. The dimension of the feature subset obtained by MQSVM is significantly lower than that of Relief-QPSO because MQSVM uses MIC to process irrelevant and redundant features, which reduces the search space in the second stage. In terms of the mean value of the results, the proposed method has the smallest value, indicating that the dimension of the feature subset obtained by the proposed method is lower than that of the existing methods.

**Table 8:** Dimensions of the feature subset.

Feature	Dimensionality	MQSVM	ReliefF-QPSO	MRMR	Fscore	Fscore-MIC	RFINCA
CO	95	**11.40**	22.00	37.20	27.00	27.00	19.60
APEN	95	**11.80**	25.00	22.20	25.80	25.80	15.20
FD	95	**11.40**	17.20	34.20	30.00	30.00	18.40
Hjorth	285	**27.00**	105.20	38.40	66.00	65.40	37.60
PSD	95	**13.80**	48.60	34.80	45.60	32.40	44.40
Fusion	665	**46.2**	242.5	103.6	121.8	117.2	103
Mean	221.67	**20.27**	76.75	45.07	52.70	49.63	39.70

Bold values indicate the smallest dimension of the feature subset.

Table 9 shows the classification accuracy. It can be found that the classification accuracy of MQSVM is not much different from the existing methods, so it is difficult to judge the performance of these methods from the classification accuracy alone.

**Table 9:** Classification accuracy.

Feature	MQSVM	ReliefF-QPSO	MRMR	Fscore	Fscore-MIC	RFINCA
CO	69.97	**70.91**	68.82	69.61	69.61	70.69
APEN	74.28	74.07	75.07	**75.72**	**75.72**	74.93
FD	82.55	82.62	**84.70**	84.20	84.20	82.55
Hjorth	**94.62**	90.68	89.60	93.62	**94.62**	93.91
PSD	97.49	97.34	96.55	**97.84**	96.84	96.77
Fusion	99.28	**99.64**	98.92	96.76	95.68	98.20
Mean	**86.36**	85.88	85.61	86.29	86.11	86.17

Bold values indicate the highest classification accuracy.

The weighted sum of the dimension and classification accuracy of the feature subset was calculated according to Eq. (28) to analyze the performance of different methods. The results are shown in Table 10. It can be found that MQSVM has significantly higher weighting values on multiple EEG features (CO, FD, Hjorth, PSD, fusion) than existing methods. At the same time, MQSVM has the largest value in terms of the mean value of the results. The above analysis shows that the feature subset obtained by the proposed method has higher classification accuracy and lower feature dimensionality than the existing methods.

**Table 10:** The weighted sum of classification accuracy and dimension of the feature subset.

Feature	MQSVM	ReliefF-QPSO	MRMR	Fscore	Fscore-MIC	RFINCA
CO	**71.78**	71.50	68.02	69.81	69.81	71.56
APEN	75.61	74.03	75.23	75.43	75.43	**75.84**
FD	**83.09**	82.55	82.63	82.62	82.62	82.35
Hjorth	**94.25**	88.20	89.32	93.02	93.04	93.27
PSD	**96.28**	92.49	93.23	93.26	93.74	92.42
Fusion	**98.66**	96.03	97.47	95.25	94.35	96.83
Mean	**86.61**	84.13	84.32	84.90	84.83	85.38

Bold values indicate the largest weighted values of feature subset dimension and classification accuracy.

The average running time of the proposed method is 227 min, which is significantly lower than the existing hybrid feature selection method, indicating that the computational complexity of the proposed method is not high. At the same time, the running time of the proposed method gently increases with the feature dimension, which indicates that the proposed method performs well on high-dimensional datasets. The dimension of the feature subset obtained by the proposed method is lower than that of the existing method, and the classification accuracy of the feature subset obtained by the proposed method is not much different from that of the existing method, which indicates that the feature subset obtained by the proposed method not only has a lower dimension but also has high classification accuracy. The weighted sum of the dimensionality and classification accuracy is computed, which facilitates a direct comparison of the performance of the feature subsets obtained by different methods. The weighted value of the proposed method is higher than that of the existing methods, which indicates that the performance of the feature subset obtained by the proposed method is better than that of the existing methods. At the same time, the weighted values of the proposed method are larger than those of the existing methods in many EEG features, which indicates that the performance of the proposed method is relatively stable across different EEG features. The above analysis shows that the feature subsets obtained by the proposed method have low dimensionality, high classification accuracy, and low computational complexity compared to the existing methods, which validates the effectiveness of the proposed method.

### Analyze the robustness of the proposed method

To further validate the robustness of the proposed method, the UCI dataset is used for experimental validation. The details of the datasets are given in Table 11. The UCI dataset is divided into a training set and a validation set; the training set is 75 percent, and the validation set is 25 percent. The parameter settings for the algorithm are the same as those used for the experiments in Section “Comparison with existing methods”.

**Table 11:** Details of the UCI dataset.

Dataset	Instance	Feature	Class
Glass	214	9	7
Heart	270	13	2
Segment	210	19	7
Dermatology	366	34	6
Ionosphere	351	34	2
Sonar	208	60	2
LSVT	126	310	2
Parkinson	756	752	2
CNAE	1080	856	9
QSAR	1687	1024	2

The weighted sum of the dimension and classification accuracy of the feature subset is calculated according to Eq. (28), and the results are shown in Table 12. It can be found that the weighted value of the proposed method is significantly higher than that of the existing method on multiple datasets (segment, dermatology, Parkinson, CNAE, QSAR) and has obvious superiority on high-dimensional datasets (Parkinson, CNAE, QSAR). By averaging the results over all datasets, it can be found that the mean value of MQSVM is significantly higher than that of the existing methods. The larger the weighted value, the better the overall performance of the feature subset. The above analysis shows that the proposed method has stable performance on datasets with different dimensions, and the extracted feature subsets perform well, which indicates that the proposed method is robust and can be used for feature extraction on other data.

**Table 12:** Weighted sum of all methods on the UCI dataset.

Dataset	MQSVM	ReliefF-QPSO	MRMR	Fscore	Fscore-MIC	RFINCA	PCA
Glass	72.17	71.98	73.82	66.57	66.57	**73.91**	70.15
heart	77.83	73.57	**85.19**	77.83	77.83	70.48	72.12
segment	**90.37**	88.33	88.60	78.97	88.42	90.28	68.11
dermatology	**95.83**	92.68	83.05	95.73	95.73	93.92	73.84
ionosphere	92.35	**95.96**	93.03	89.76	89.76	93.20	94.69
sonar	78.97	**80.85**	72.75	69.22	69.22	70.95	82.51
LSVT	90.94	**93.95**	82.92	91.43	91.43	82.89	77.36
Parkinson	**90.04**	89.78	86.64	74.39	74.39	77.24	77.25
CNAE	**88.91**	77.48	74.96	69.01	69.01	74.26	88.17
QSAR	**90.41**	87.60	88.68	89.09	89.09	89.72	86.72
mean	**86.78**	85.22	82.96	80.20	81.14	81.68	79.09

Bold values indicate the largest weighted values of feature subset dimension and classification accuracy.

## Conclusions

In this paper, we propose a hybrid EEG feature selection method to reduce the dimensionality of EEG features and improve classification accuracy. MIC is used to eliminate irrelevant features and redundant features, which effectively reduces the search space in the second stage and improves the speed of the method. In the second stage of feature selection, QPSO is used to optimize a subset of features, which avoids the problem that conventional PSO quickly gets stuck in local optima. Considering the coupling between the feature subset and the classifier, QPSO is used to optimize the parameters of the classifier while optimizing the feature subset, which increases the classification accuracy and reduces the computational complexity of the algorithm. Several EEG features of different dimensions are extracted from a public EEG dataset, and the proposed method is compared with five existing feature selection methods. Experimental results show that the feature subsets obtained by the proposed method have low dimensionality, high classification accuracy, and low computational complexity. Thus, the proposed method has a clear advantage over existing EEG feature selection methods.

Despite the beneficial results obtained in this study, there are some limitations. First, the proposed method uses MIC to measure feature performance to eliminate irrelevant and redundant features. However, MIC suffers from high computational complexity and long running time when the dimensionality of the dataset is high. Although mutual information and the Pearson correlation coefficient can measure correlations, mutual information is challenging to remove redundant features, and the Pearson correlation coefficient is difficult to measure nonlinear relationships between features. Future work will involve identifying and proposing a low computational complexity correlation measure to reduce the overall computational complexity of the algorithm. Moreover, the QPSO used in the proposed method may still get stuck in local optimality. Therefore, future work can focus on improving the optimization algorithm to improve the global search capability of the algorithm.

## Data Availability

The UCI datasets analysed during the current study are available in the [UC Irvine Machine Learning Repository] repository, [http://archive.ics.uci.edu/datasets]. The EEG dataset analysed during the current study are available in the [http://figshare.com/] repository, [https://figshare.com/articles/EEG_Data_New/4244171].
